# Efficacy of 27-Gauge Vitrectomy with Internal Limiting Membrane Peeling for Epiretinal Membrane in Glaucoma Patients

**DOI:** 10.1155/2019/7807432

**Published:** 2019-12-13

**Authors:** Masaaki Yoshida, Hiroshi Kunikata, Shiho Kunimatsu-Sanuki, Toru Nakazawa

**Affiliations:** ^1^Department of Ophthalmology, Tohoku University Graduate School of Medicine, Sendai, Japan; ^2^Department of Retinal Disease Control, Tohoku University Graduate School of Medicine, Sendai, Japan; ^3^Department of Advanced Ophthalmic Medicine, Tohoku University Graduate School of Medicine, Sendai, Japan; ^4^Department of Ophthalmic Imaging and Information Analytics, Tohoku University Graduate School of Medicine, Sendai, Japan

## Abstract

**Purpose:**

To evaluate the efficacy of epiretinal membrane (ERM) surgery for patients with ERM and glaucoma.

**Methods:**

We reviewed the medical records of 20 consecutive ERM patients with glaucoma, who underwent 27-gauge microincision vitrectomy surgery (27GMIVS) with internal limiting membrane (ILM) peeling. The preoperative and 6-month postoperative visual field was tested with the Humphrey Field Analyzer (HFA) 24-2 program. Changes in threshold sensitivity in the HFA test points were analyzed point-by-point, with points classified into groups based on sensitivity as abnormal (less than 5th percentile in pattern deviation) or normal (all other points) and based on location as central (12 central points) or peripheral (all other points) with a linear mixed-effects model.

**Results:**

Visual acuity and mean deviation improved postoperatively (*P* < 0.001 for both) in all patients. Threshold sensitivity in central or peripheral points that were abnormal preoperatively improved postoperatively (*P*=0.006 or *P* < 0.001, respectively). Threshold sensitivity also improved in the central normal test points (*P*=0.03), but not in the peripheral normal points (*P*=0.12).

**Conclusion:**

Visual acuity improved, and there was no visual field progression, after ERM surgery in glaucomatous eyes during a 6-month postoperative follow-up, suggesting that ERM and ILM removal using 27GMIVS may be effective even in glaucomatous eyes.

## 1. Introduction

An epiretinal membrane (ERM) is a thin layer of fibrous tissue that can form on the inner surface of the central retina, causing metamorphopsia, monocular diplopia, and decreased vision [1–3]. Recent advances in microincision vitrectomy surgery (MIVS) and ERM removal with internal limiting membrane (ILM) peeling have improved surgical safety and visual outcomes for ERM patients [4–7]. However, ILM peeling is still a difficult technique and can inflict mechanical stress on the central retina, including the nerve fiber layer and ganglion cell layer [8–10]. These layers are also affected by glaucoma, which is associated with thinning of the retinal nerve fiber layer (RNFL) and the death of retinal ganglion cells (RGCs), causing visual field defects [11, 12]. Thus, the effects of vitrectomy with ERM and ILM peeling on the visual field in glaucomatous eyes are of particular concern, especially considering that both ERM and glaucoma become more common with age and many countries are now affected by aging populations.

Previously, only two studies have examined the effects of vitrectomy for ERM or macular hole (MH) on the visual field in patients with glaucoma [13, 14]. Despite these reports, it is still unclear whether the risks of ERM surgery are acceptable in patients with glaucoma, even when MIVS is used. Thus, this study set out to determine whether glaucoma patients who received ERM and ILM peeling underwent accelerated progression of existing visual field defects during the first six months postoperatively. To test this, we evaluated visual field sensitivity changes in patients with ERM and glaucoma, in visual field test points that were divided into abnormal and normal groups, after 27-gauge MIVS (27GMIVS) with ILM peeling.

## 2. Materials and Methods

### 2.1. Subjects

This retrospective study was approved by the Institutional Review Board of Tohoku University and was conducted in accordance with the tenets of the Declaration of Helsinki. A consecutive series of glaucoma patients that underwent 27GMIVS for ERM at Tohoku University Hospital from June 2016 to March 2017 were enrolled. Visual field testing was performed with the Humphrey Field Analyzer (HFA) 24-2 Swedish interactive threshold algorithm (Carl Zeiss Meditec Inc., Dublin, CA, USA). The inclusion criteria were (1) a diagnosis of idiopathic ERM and (2) a diagnosis of open-angle glaucoma with a visual field meeting the Anderson-Patella classification [15]. Exclusion criteria were (1) macular disease other than ERM, (2) previous vitreoretinal or glaucoma surgery, and (3) rates of HFA 24-2 fixation loss, false positives, or false negatives greater than 20%. Twenty eyes of 20 patients met all criteria.

### 2.2. Surgical Procedures

**Figure 1:**
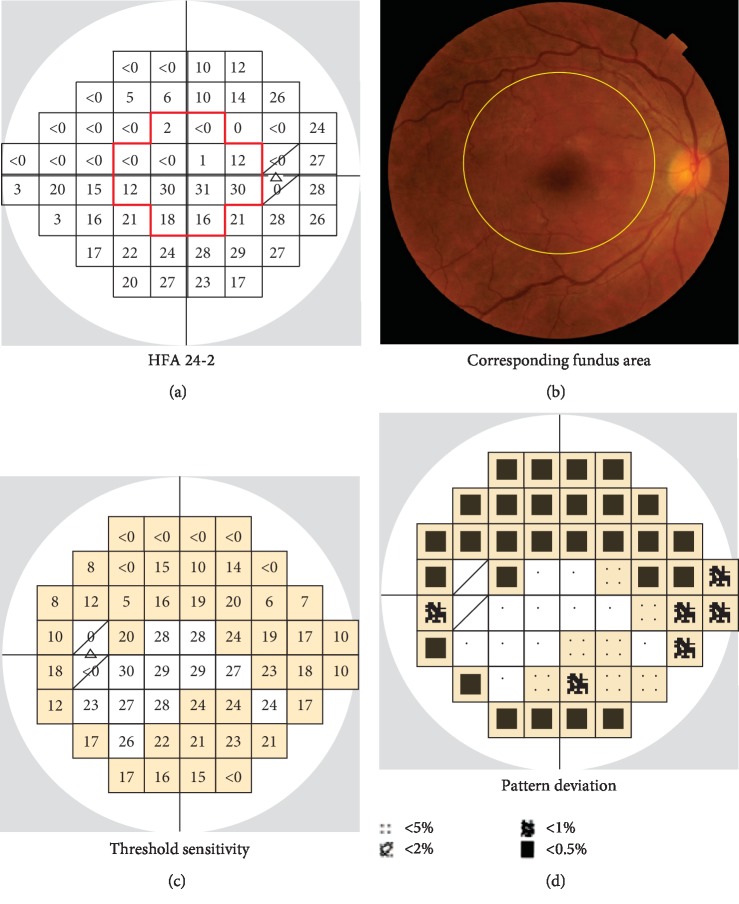
(a) Illustrative example of Humphrey Field Analyzer (HFA) 24-2 test results from a right eye. The 24-2 program has 52 test points, excluding 2 test points (shown by slashes) corresponding to Mariotte's blind spot. We divided the 52 test points into central points (the 12 points within the red line) and peripheral points (the other 40 points). (b) The area of the fundus corresponding to the HFA 24-2 test points. The ILM was peeled from a circular area, with a radius approximately equal to 2 disc diameters, surrounding the macula (shown by the yellow line) and corresponding to the 12 central test points. (c) Illustrative example of HFA 24-2 results from a left eye. According to findings for pattern deviation (d), the test points were classified as normal or abnormal if they had a sensitivity of more than or less than the 5th percentile (shown in yellow), respectively.

All surgeries were performed by a single surgeon (HK). All patients older than 50 years with phakic eyes underwent cataract surgery in combination with MIVS. All patients younger than 50 years with phakic eyes, as well as all patients with pseudophakic eyes, underwent only MIVS. The procedure was based on standard 3-port PPV, using 27-gauge instruments and the Constellation Vision System (Alcon Surgical, Fort Worth, TX, USA). After resecting the vitreal core, about 4 mg of triamcinolone acetonide (TA; MaQaid, Wakamoto Pharmaceutical, Tokyo, Japan) was injected into the vitreous cavity to determine whether a posterior vitreous detachment (PVD) was present. If a PVD was not present, a PVD was created by suction with a vitreous cutter. After creating a PVD and removing peripheral residual gel, the ERM and ILM were completely peeled and then removed using an intraocular end gripping forceps, assisted by TA. A circular section of the ILM with a radius approximately equal to 2 disc diameters was removed from around the macula, as shown in Figure 1. The initial ILM flap was normally made on the temporal side, but if the retina had a region with zero threshold sensitivity within the section of ILM to be removed, the initial flap was made in that region, according to the surgeon's judgement. We did not use any dye to visualize the ILM during the ILM peeling procedure. Fluid-gas exchange was not performed and expanding gas was also not injected at the end of the surgery.

### 2.3. Statistical Analysis of Visual Field Test Points and Other Clinical Data

**Figure 2:**
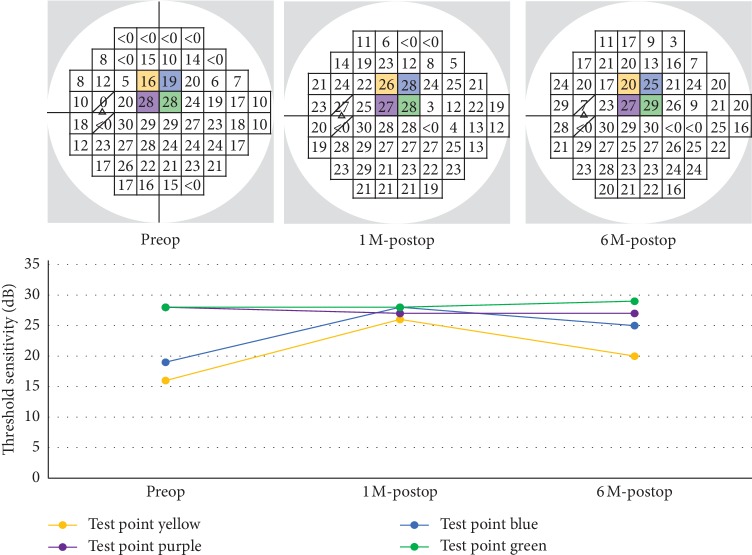
Analysis of postoperative changes in threshold sensitivity in 4 representative test points, using dB values as an example. At the top, preoperative, 1-month, and 6-month HFA 24-2 results from a single patient are shown from left to right, respectively. Postoperative changes in threshold sensitivity in each test point (shown in yellow, blue, purple and green) are shown in the graph at the bottom.

Visual field testing with the HFA 24-2 program was performed preoperatively and at 1 month and 6 months postoperatively. The HFA 24-2 program has 52 test points, excluding 2 test points that correspond to Mariotte's blind spot. The 12 central test points approximately correspond to the section of the ILM that was peeled (Figure 1). Thus, we divided the 52 test points into central points, comprising the central 12 points, and peripheral points, comprising the other 40 points, as shown in Figure 1. Separately, we divided the 52 test points into “abnormal” and “normal” points according to results for pattern deviation in preoperative HFA 24-2 testing (abnormal: sensitivity less than the 5th percentile; normal: all other points). Test points with 0 dB threshold sensitivity were excluded from the analysis. Then, we recorded postoperative changes in threshold sensitivity in each test point (as shown in Figure 2) and calculated the slope for each of the above four types of point with a linear mixed-effects model using the statistical computing software R, version 3.4.2, with the lmerTest package [16, 17]. The difference in slope between the different types of points was also estimated and tested with the linear mixed-effects model. These analyses were performed for dB and 1/Lambert (1/L) values (antilogged values of dB; 1/L = 10^dB/10^). Data from left eyes were flipped to match the right eyes. Postoperative changes in best-corrected visual acuity (BCVA), intraocular pressure (IOP), optical coherence tomography (OCT) parameters, and other HFA 24-2 parameters (i.e., mean deviation and foveal threshold) were also evaluated with a linear mixed-effects model. OCT parameters included the thickness of the macular retinal nerve fiber layer (mRNFL), circumpapillary retinal nerve fiber layer (cpRNFL), ganglion cell layer + inner plexiform layer (GCIPL), and mRNFL + GCIPL (GCC), measured with 3D OCT 2000, version 8.11 (Topcon Corporation, Tokyo, Japan). The thickness of the mRNFL, GCIPL, and GCC was recorded as averages of measurements in a 6 × 6 mm area in the macula. *P* values <0.05 were considered statistically significant.

## 3. Results

Clinical background data for 20 eyes of 20 patients are shown in Table 1. The average age was 68.9 years. 27GMIVS with cataract surgery was performed in 18 eyes and without cataract surgery in 2 eyes (lens-sparing surgery was performed in one case; the other eye was pseudophakic preoperatively). Thirteen patients (65%) had normal-tension glaucoma, and 4 patients (20%) had primary open-angle glaucoma. Three patients (15%) had unclassified open-angle glaucoma. The average mean deviation (MD) in preoperative HFA 24-2 testing was −8.2 dB. Seven, 8, and 5 eyes had early, moderate, and advanced glaucoma, respectively. The total group of test points comprised 1040 points (52 test points in each of 20 eyes), including 571 normal test points and 345 abnormal test points. There were 124 test points with 0 dB preoperative sensitivity. Among the 240 central test points (12 test points in each of the 20 eyes), there were 160 normal points and 80 abnormal points, including 22 central test points with 0 dB preoperatively. Though 25 of 240 test points in the central area, i.e., the ILM-peeled area, showed some deterioration in postoperative threshold sensitivity, this did not constitute significant visual field progression.

Table 2 shows preoperative and postoperative parameters. BCVA significantly improved postoperatively (*P* < 0.001). The average preoperative IOP was 13.5 mmHg (including patients who did or did not use topical antiglaucoma medications); IOP did not change significantly postoperatively (*P*=0.07). Among OCT findings, the thickness of the temporal cpRNFL (i.e., in the temporal quadrant), mRNFL, GCIPL, and GCC significantly decreased postoperatively (*P*=0.04, *P* < 0.001, *P*=0.01, and *P* < 0.001, respectively) while the thickness of the overall cpRNFL did not change (*P*=0.68). Among HFA findings, both MD and foveal threshold (in both dB and 1/L values) significantly improved postoperatively (*P* < 0.01, *P*=0.01 and *P*=0.04, respectively). Although threshold sensitivity in the overall set of normal test points did not improve postoperatively, in either dB or 1/L values (*P*=0.09 and *P*=0.07, respectively), sensitivity did significantly improve in the overall set of abnormal test points, in both dB and 1/L values (*P* < 0.001 for both). No patients experienced any postoperative adverse events such as rhegmatogenous retinal detachment or endophthalmitis.

**Figure 3:**
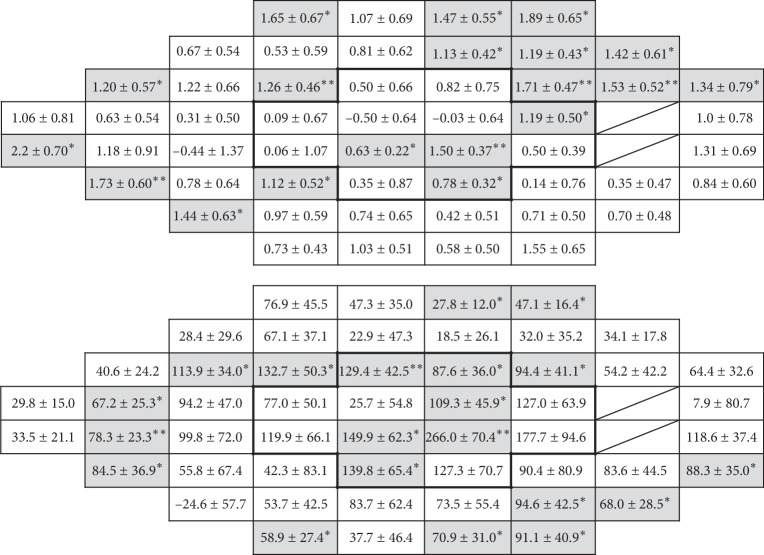
Local distribution of the slope of postoperative changes in threshold sensitivity measured with HFA 24-2. Maps of the slopes are shown oriented to right-eye visual field. Results in dB values and 1/Lambert values are shown at the top and the bottom, respectively. The 12 central test points are shown by the bold lines. Mariotte's blind spot is shown with slashes. ^*∗*^*P* < 0.05; ^*∗∗*^*P* < 0.01.

Figure 3 shows, as maps, the slope of threshold sensitivity changes in each of the 52 test points (oriented to match the right eyes). Threshold sensitivity did not significantly deteriorate in any of the test points, while 19 of 52 test points (37%) showed an improvement in threshold sensitivity in dB values and 20 of 52 test points (38%) showed an improvement in 1/L values. The test points showing improved threshold sensitivity did not follow any specific pattern.

**Figure 4:**
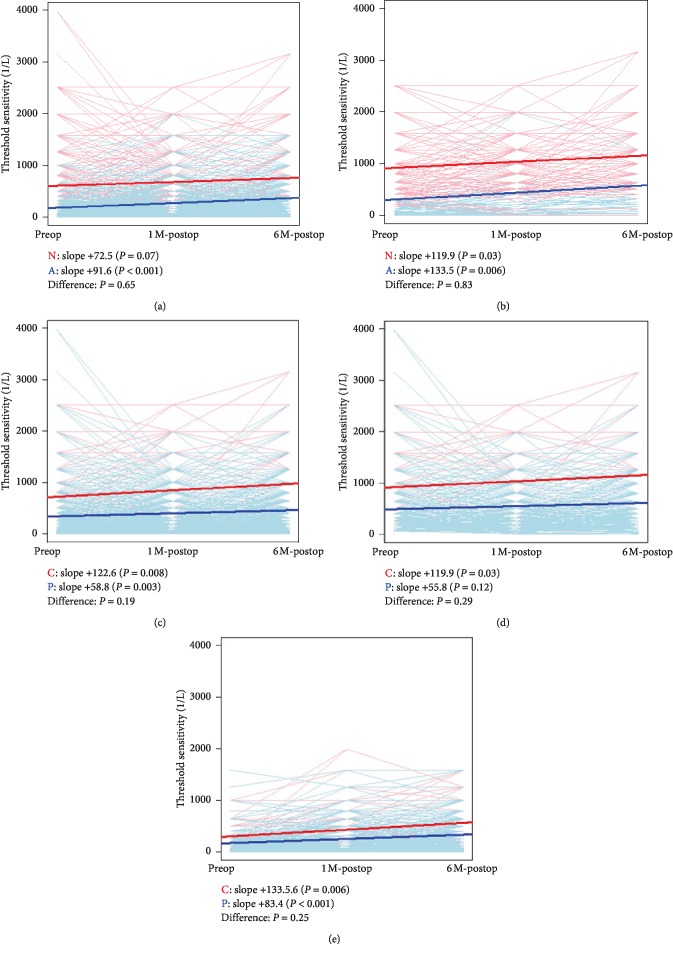
Analysis of postoperative changes in threshold sensitivity with a linear mixed-effects model, using 1/Lambert (1/L) values. (a) Analysis of all test points in the entire measurement area. Comparison between normal test points (red) and abnormal test points (blue). (b) Analysis of test points in the central area. Comparison between normal test points (red) and abnormal test points (blue). (c) Analysis of all test points in the entire area. Comparison between central test points (red) and peripheral test points (blue). (d) Analysis of normal test points in the entire area. Comparison between central test points (red) and peripheral test points (blue). (e) Analysis of abnormal test points in the entire area. Comparison between central test points (red) and peripheral test points (blue). N = normal; A = abnormal; C = central; P = peripheral.

Figure 4 shows the slope in each group and statistical differences in slope between different types of points (1/L values are shown). Figures 4(a) and 4(b) show results for the normal test points and abnormal test points.  Figure 4(a) shows results for the entire HFA visual field, and Figure 4(b) shows results for the central area only. Although the normal test points in the entire field did not show an improvement in threshold sensitivity (*P*=0.07), the other types of points, i.e., the abnormal test points in the entire field and central area and the normal test points in the central area, showed a significant improvement (*P* < 0.001, *P*=0.006, and *P*=0.03, respectively). Differences in slope between the normal test points and the abnormal test points were not significant when analyzing either the entire field or the central area by itself. Results of analyses of the central area and the peripheral area are shown in Figures 4(c)–4(e). Figures 4(c) and 4(e), showing results from all test points and the abnormal test points, respectively, show that both the central test points and the peripheral test points significantly improved, without a significant difference between them. However, among the normal test points (Figure 4(d)), only the central points significantly improved (*P* = 0.03), while the peripheral test points did not (*P* = 0.12), although the difference between them was not statistically significant.

## 4. Discussion

Previously, there have been two reports describing the effects of vitrectomy in macular diseases, including ERM, in glaucomatous eyes [13, 14]. The report by Moroi et al. was a small case series (ERM: 5 eyes, MH: 2 eyes; total: 7 eyes) that showed an improvement in visual acuity of at least 2 lines in 5 of 7 eyes after 20-gauge pars plana vitrectomy (PPV) without combined cataract surgery [13]. Although MD significantly decreased postoperatively, this decrease was consistent with previous measurements of glaucomatous progression. Moreover, new visual field defects did not emerge. Thus, Moroi et al. concluded that vitrectomy was usable in eyes with glaucoma and coexisting macular problems. On the other hand, a study by Tsuchiya et al. found that mean sensitivity in the central area (within 10° eccentricity) decreased postoperatively, while it increased in the peripheral area, in an analysis of 54 eyes (ERM: 42 eyes, MH: 12 eyes) followed for an average of 10.3 months [14]. MIVS in that study used various instrument gauges, including 23-, 25-, and 27-gauge. Tsuchiya et al. concluded that vitrectomy for ERM or MH had a negative effect on the visual field in eyes with glaucoma.

Compared to these previous studies, our study had important advantages. First, we included only cases of ERM. Second, all procedures were performed with 27GMIVS, with only TA as an adjuvant, by the same expert vitreous surgeon. Third, we compared retinal sensitivity changes not only between the central and peripheral areas but also between normal and abnormal test points, i.e., regions affected or unaffected by glaucoma. Our results thus reliably show that visual acuity improved, without visual field progression, after ERM surgery in the glaucomatous eyes included here during a 6-month postoperative follow-up.

Various intraoperative factors can increase retinal damage caused by vitrectomy surgery and affect the postoperative visual field. Previous studies investigating the effects of macular surgery in glaucomatous eyes included cases not only with ERM but also with MH [13, 14]. However, we consider that these diseases cannot be studied together because of critical differences in the surgical procedures used to treat them. Fluid-air exchange, which is routinely used in MH surgery, can cause postoperative visual field defects [18–22]. Furthermore, the use of an expanding gas, such as sulfur hexafluoride (SF6) or perfluoropropane (C3F8), at the end of surgery can cause a postoperative elevation in IOP, leading to secondary glaucoma [23, 24]. Finally, dye-assisted ILM peeling can cause long-term visual field defects after macular surgery [21, 22]. Thus, to isolate the effect of ILM peeling on the macular region in glaucomatous eyes, we included only cases that underwent ERM surgery without using any dye, thereby excluding the above confounding factors. Furthermore, though MIVS with any instrument gauge is considered minimally invasive and reduces postoperative inflammation and IOP instability, we chose to use 27GMIVS to reduce retinal damage as much as possible [25–28].

In the current study, 18 of 20 cases underwent 27GMIVS combined with cataract surgery. Though all cases of cataract were classified as having a grade 2 or lower lens nucleus in the Emery-Little classification, the improvements in threshold sensitivity was similar in the central and peripheral test points (Figure 4(c)). Thus, it is possible that the cataract surgery caused the postoperative increase in threshold sensitivity we observed in both types of test point. However, this possibility is contradicted by our analysis of the normal test points, which showed that only the central test points significantly improved, while the peripheral test points did not (Figure 4(d)). Thus, improved sensitivity in the central test points was also likely due to the ERM and ILM peeling, not only the cataract surgery. It is possible that the normal test points in the peripheral area did not improve, even though the abnormal test points did, because the normal points do not usually have enough potential to improve further postoperatively because they have high threshold sensitivity preoperatively, i.e., there is a saturation effect. Nonetheless, even normal test points in the central area might have the potential to improve after surgery due to preoperative decreased threshold sensitivity caused by ERM.

Interestingly, the current study found that there were no visual field test points in the glaucomatous eyes in which retinal sensitivity significantly decreased after ERM surgery. Two HFA 24-2 test points, both located in the nasal area and corresponding to the temporal quadrant of the macula, have previously been reported to show significant and sustained deterioration after macular surgery for ERM or MH in glaucomatous eyes [14]. Generally, the nasal visual field is prone to complications, such as paracentral scotomas and decreased retinal sensitivity, after ILM peeling for MH, even in nonglaucomatous eyes [19, 29]. In the current study, the temporal quadrant also seemed to fare relatively worse postoperatively; threshold sensitivity increased in many points outside the temporal quadrant, but none in the temporal quadrant. This may be due to specific anatomical characteristics of the temporal quadrant, including a thinner RNFL [30], the presence of the temporal raphe, i.e., the watershed zones [31], and the common practice of making the initial ILM flap in the temporal quadrant, which might induce direct mechanical stress [9]. In the current study, we modified the ILM peeling procedure by creating the initial ILM flap in an area of the retina with zero sensitivity if such an area was present. Although we cannot make definite conclusions about the impact of our approach to initial flap position because this study was noncomparative and had a small sample size, we assume that the per-patient variation in initial flap position might have reduced the unwanted direct mechanical stress on active RGCs and the RNFL, thus protecting the visual field points with remaining retinal sensitivity.

This study was the first to separately investigate normal and abnormal test points in eyes with both glaucoma and ERM. We considered that this approach might reveal important new information, because we hypothesized that the abnormal test points, which corresponded to areas of the retina with thinning of the RNFL and GCC [32–34], might be more vulnerable to mechanical stress and thus more prone to deterioration than the normal points. However, threshold sensitivity in the central test points, i.e., the area from which the ERM and ILM are peeled, improved in the abnormal points, and this finding was confirmed in 1/L values (i.e., in linear scale). Moreover, there were no differences in slope in the normal and abnormal points in the central area. This suggests that less-invasive ERM surgery with ILM peeling can be effective, even in glaucomatous eyes with ERM.

The limitations of this study included a relatively short follow-up time of 6 months, a small study population of 20, the fact that all surgeries were performed by the same surgeon, and the omission of postoperative findings for other subjective measurements of retinal sensitivity, such as microperimetry, or objective measurements of retinal function, such as focal electroretinography. Furthermore, although it might have been desirable to evaluate only ERM eyes that did not also undergo cataract surgery, collecting data from such eyes is difficult in Japan, because vitrectomy is very commonly combined with cataract surgery in this country. Long-term follow-up is therefore important to evaluate the true benefits of ERM surgery. However, follow-up periods longer than one year might also introduce new difficulties in judging glaucoma progression or the incidence of ERM surgery-related damage.

In conclusion, this study showed that during a 6-month follow-up period after TA-assisted 27GMIVS for ERM in glaucomatous eyes, BCVA improved and there was no visual field progression. Threshold sensitivity in the central area of the retina, corresponding to the area of ILM peeling, improved in abnormal HFA test points of the eyes, as well as in the normal test points. Thus, TA-assisted ERM surgery based on 27GMIVS can be considered effective, even in eyes with glaucoma.

## Figures and Tables

**Table 1:** Clinical background of glaucomatous patients with epiretinal membrane.

Sex: male/female	6/14
Age (YO)	68.9 ± 8.1
Laterality: right/left	4/16
Axial length (mm)	24.5 ± 1.5
Operation	
27GMIVS with cataract surgery	18/20
27GMIVS in pseudophakic eye	1/20
Lens-sparing 27GMIVS	1/20
Type of glaucoma	
Normal tension glaucoma	13/20
Primary open-angle glaucoma	4/20
Unknown (open-angle glaucoma)	3/20
Disease severity	
Average MD (dB)	−8.7
Early (MD *>* −6)	7/20
Moderate (−12 ≤ MD ≤ −6)	8/20
Advanced (MD *<* −12)	5/20
Test points (all)	
Normal	571/1040
Abnormal	345/1040
0 dB	124/1040
Test points (central)	
Normal	160/240
Abnormal	58/240
0 dB	22/240

MD: mean deviation of Humphrey Field Analyzer 24-2 program; 27GMIVS: 27-gauge microincision vitrectomy surgery. The numbers of all test points and central test points are 1040 and 240, respectively, because each eye has 52 test points and 12 central test points.

**Table 2:** Preoperative and postoperative parameters.

	Preop	1 M-postop	6 M-postop	Slope	*P* value
BCVA (logMAR)	0.29	0.088	0.032	−0.13	<0.001
IOP (mmHg)	13.5	13.2	12.7	−0.50	0.07
OCT findings					
cpRNFL (total) (*μ*m)	93.4	99.1	94.5	+0.55	0.68
cpRNFL (temporal) (*μ*m)	94.3	92.2	85.9	−4.2	0.04
mRNFL (*μ*m)	48.1	37.7	37.4	−5.4	<0.001
GCIPL (*μ*m)	70.6	62.9	61.5	−4.5	0.001
GCC (*μ*m)	118.7	100.6	98.9	−9.9	<0.001
HFA findings					
MD (dB)	−8.7	−7.9	−6.7	+1.0	<0.001
Foveal threshold (dB)	31.9	32.3	33.6	+0.88	0.01
Foveal threshold (1/L)	1895.3	2112.7	2607.1	+355.9	0.04
Threshold sensitivity					
Normal test points (dB)	26.9	26.9	27.7	+0.38	0.09
Abnormal test points (dB)	17.4	19.0	20.8	+1.6	<0.001
Normal test points (1/L)	654.6	669.4	798.0	+72.5	0.07
Abnormal test points (1/L)	148.8	266.3	327.9	+91.6	<0.001

Linear mixed-effects model. BCVA: best-corrected visual acuity; IOP: intraocular pressure; OCT: optical coherence tomography; cpRNFL; circumpapillary retinal nerve fiber layer thickness; mRNFL: macular retinal nerve fiber layer thickness; GCIPL: ganglion cell layer thickness+ inner plexiform layer thickness; GCC: ganglion cell complex thickness (mRNFL + GCIPL); HFA: Humphrey Field Analyzer; MD: mean deviation.

## Data Availability

The data used to support the findings of this study cannot be made freely available. Requests for access to these data should be made to Dr. Masaaki Yoshida (e-mail: masaaki@oph.med.tohoku.ac.jp).
